# Association between self-reported sleep apnea and biomarkers of liver injury: Evidence from National Health and Nutrition Examination Survey

**DOI:** 10.1097/MD.0000000000039393

**Published:** 2024-09-06

**Authors:** Yi-Bin Jiang, Zhi-Wei Huang, Xue-Jun Lin, Jia-Min Luo, Li-Da Chen

**Affiliations:** a Health Management Center, Zhangzhou Affiliated Hospital of Fujian Medical University, Zhangzhou, Fujian Province, People’s Republic of China; b Department of Otolaryngology, Quanzhou First Hospital Affiliated to Fujian Medical University, Quanzhou, Fujian Province, People’s Republic of China; c Department of Laboratory Medicine, Zhangzhou Affiliated Hospital of Fujian Medical University, Zhangzhou, Fujian Province, People’s Republic of China; d Department of Respiratory and Critical Care Medicine, Zhangzhou Affiliated Hospital of Fujian Medical University, Zhangzhou, Fujian Province, People’s Republic of China.

**Keywords:** cross-sectional survey, liver injury, NHANES, nonalcoholic fatty liver disease, sleep apnea

## Abstract

The community population based studies on the relationship between obstructive sleep apnea and liver injury are limited. The study aimed to clarify the association between sleep apnea (SA) and liver injury by using the data in The National Health and Nutrition Examination Survey. SA was assessed by the sleep questionnaire and liver injury was evaluated by liver function test, hepatic steatosis index, and fibrosis-4. Weighted multivariable linear regression was performed to examine the association between SA and liver injury. Subgroup analyses and sensitivity analysis were also conducted. A total of 19,362 eligible participants were included in the study. After adjusting for confounders, the presence of SA was significantly associated with increased levels of lnALT, lnAST/alanine aminotransferase, lnGGT, and lnHSI (all *P* values < .05), but not with lnFIB-4 (*P* > .05). There is a dose–response relationship between the severity of SA and increased levels of lnALT, lnGGT, and decreased levels of lnAST/alanine aminotransferase (test for trend, all *P* values < .05). Subgroup analyses revealed that the positive association between SA and liver function, liver steatosis showed a tendency to exist in nonobese, younger, non-Hispanic Black, and male populations. Sensitive analysis showed the relationship between SA and liver injury was stable. Self-reported SA was independently associated with elevated liver enzymes and liver steatosis among US population. The association was more pronounced among nonobese, younger, non-Hispanic Black, and male populations.

## 1. Introduction

Obstructive sleep apnea (OSA) is a highly prevalent condition linked to numerous adverse health outcomes, including cardiovascular problems,^[1]^ cognitive impairment,^[2]^ metabolic disorder,^[2]^ and an increased risk of traffic accidents.^[3]^ The prevalence of OSA in the overall population ranges from 9% to 38%.^[4]^ Clinically, the OSA patients exhibit a recurring pattern of upper airway obstruction and frequent awakenings during sleep, resulting in chronic intermittent hypoxia (CIH), sleep fragmentation, daytime sleepiness, and even hypercapnia. The most common risk factors of OSA are age, male gender, and obesity.^[5]^ Race difference represents another risk factor for OSA.^[6]^

Over the last 2 decades, there has been a growing interest in the correlation between OSA and liver injury, particularly nonalcoholic fatty liver disease (NAFLD).^[7,8]^ We previously included 85 patients with NAFLD and found a significant independent correlation between OSA-related hypoxia and the biochemical markers of liver injury.^[9]^ A recent study analyzing 183 obesity patients demonstrated that OSA exacerbated NAFLD and increased the risk of nonalcoholic steatohepatitis in obese patients based on liver histopathological evidence.^[10]^ However, most of studies exploring this issue were performed in a clinically based setting and limited by small sample size. The community population based studies on the relationship between OSA and liver injury are lacking.

Thus, we designed this study to clarify the association between sleep apnea (SA) and liver injury by using the large community-based data in The National Health and Nutrition Examination Survey (NHANES). Furthermore, the subgroup analyses were subsequently conducted to explore this association according to age, body mass index (BMI), gender, and race.

## 2. Methods

### 2.1. Study population

The NHANES is a complex, nationally representative, stratified, multistage probabilistic health survey of the US population conducted every 2 years since 1999 by the Centers for Disease Control and Prevention. The NHANES study was approved by the Ethical Review Board of the National Center for Health Statistics, and all participants provided informed written consent. NHANES database are freely and publicly available. The data from 2005 to 2008 and 2015 to 2020 NHANES database with complete questionnaires data on sleep disorders (snoring, gasping, and stopping breathing while sleeping) and complete liver function test were extracted in the current study. Both outcomes and exposure data were derived from all available survey cycles within this timeframe. Sleep questionnaire SLQ040 was not adopted in the 2009 to 2014 NHANES survey, leading us to exclude these specific data sets. The exclusion criteria for this study are outlined below: 1. excess alcohol intake (defined as >3 standard drinks per day in males; or >2 standard drinks per day in females)^[11]^; 2. pregnant women; 3. virus hepatitis (positive hepatitis B surface antigen or hepatitis C virus RNA); 4. age <16 years old; 5. missing data on exposures and outcomes.

### 2.2. Outcome assessment

The liver injury was evaluated by the aspects including liver function, liver steatosis, and liver fibrosis. Liver function was determined by measuring serum alanine aminotransferase (ALT), aspartate aminotransferase (AST), AST/ALT, total bilirubin, albumin, total protein (TP), and alkaline phosphatase, gamma glutamyl transpeptidase. The hepatic steatosis index (HSI) was applied to quantify the degree of liver steatosis. Liver fibrosis was estimated using the fibrosis-4 (FIB-4) index. The following formulas were used for calculating HSI and FIB-4. HSI = 8 × ALT/AST ratio + BMI (kg/m^2^) (+2, if diabetes mellitus; +2, if female). FIB-4 = (age [years] × AST [U/L])/(platelet [10^9^/L] × (ALT [U/L])^1/2^).

### 2.3. Exposure assessment

The presence and severity of SA relied solely on self-reported symptoms and were not corroborated by polysomnography. SA was judged by the individual’s responses to the questionnaire on sleep disorders (how often did you snort, gasp, or stop breathing while asleep?).^[12]^ The presence and severity of SA were based on the following answer: rarely (1–2 nights per week), occasionally (3–4 nights per week), and frequently (5 or more nights per week). The answer of never was classified as absent SA.

### 2.4. Covariates assessment

The covariates included in the models were selected a priori, in accordance with prior empirical evidence linking OSA and liver injury.^[9,13,14]^ Gender, age, BMI, race, family income-to-poverty ratio (PIR), smoking status, drink status, hypertension, diabetes, and coronary heart disease (CHD) were taken into account as potential confounding factors. Race was divided into 5 distinct categories, which included Mexican American, other Hispanic, non-Hispanic White, non-Hispanic Black, and other racial groups. BMI was calculated by weight (kg)/height (m^2^). The presence of hypertension, diabetes, and CHD was established through self-reported physician diagnoses, indicating a history of these conditions. Smoking status was classified into 2 categories: smokers and nonsmokers, based on the response to the following question: “Have you smoked at least 100 cigarettes in your lifetime?” As for drinking status: nondrinker, did not have more than 12 drinks in entire life (data from 2005–2008 and 2015–2016) or did not have 1 drink of any kind of alcohol (data from 2017–2020); excess drinker: had more than 2 drinks per day for females or more than 3 drinks per day for males; the rest was classified as drinker. The excess drinkers were excluded from this study.

### 2.5. Statistical analysis

All analyses applied Mobile Examination Center sample weights to account for oversampling, nonresponse, noncoverage, and to permit nationally representative estimates. The continuous variables were reported as the weighted mean with standard deviation, and the categorical variables were shown as counts with weighted percentages. To compare the differences between the 4 groups, Chi-square test was used for categorical variables, weighted linear regression was used for continuous variables. Weighted multivariate linear regression analyses were applied to explore the independent associations between SA and outcome variables. Tests for linear trends were also performed. A log transformation was employed for skewed outcome data. In addition, we divided subjects into subgroups based on BMI, age, gender, and race, and then investigated the relationship between SA and liver injury within each subgroup. In sensitive analysis, the missing values of confounders were managed using random forest interpolation with the “missForest” R package. The missing data of covariate data are summarized in Table S1, Supplemental Digital Content, http://links.lww.com/MD/N417. Weighted multivariate linear regression analyses adjusting the confounding variables including imputed data were further performed to examine the robust result. All analyses were performed in R software (version 4.3.0; R Foundation for Statistical Computing) and GraphPad Prism 8 (GraphPad Software Inc.). All tests were conducted with a two-tailed approach. *P* values <.05 were regarded as statistically significant.

## 3. Results

### 3.1. Baseline characteristics in subjects stratified by SA severity

**Figure 1.**
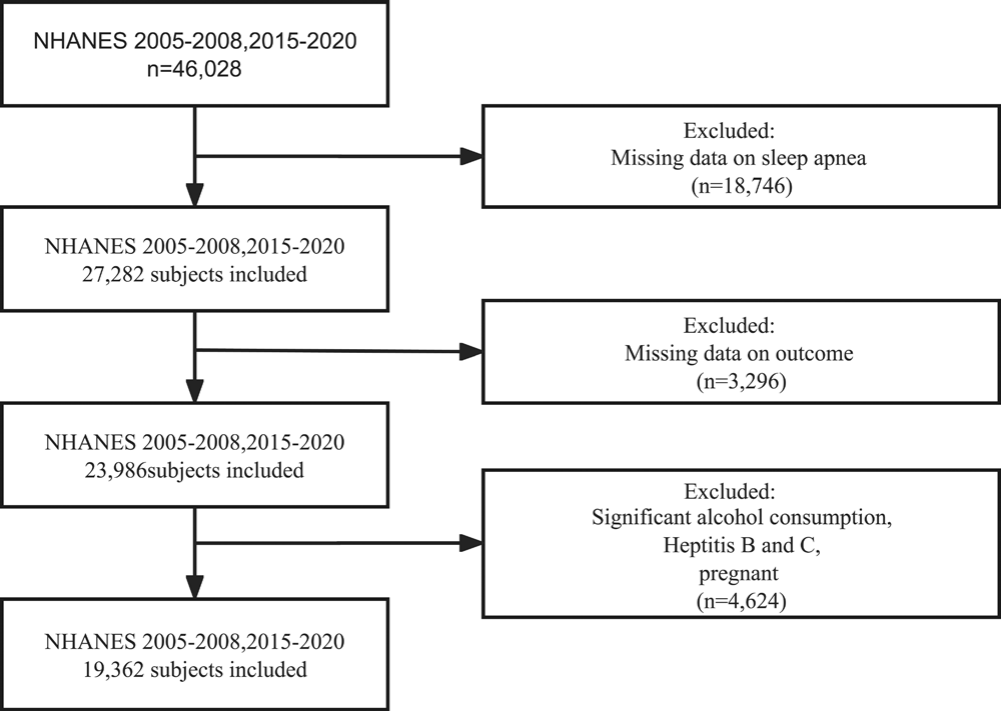
The flow chart of selection process.

A total of 19,362 eligible participants were included in the final analysis. Detailed information about the inclusion and exclusion is provided in Figure 1. Baseline characteristics and liver injury parameters stratified by SA severity are summarized in Table 1. Among the participants, 15,220 were classified as no SA, 2038 as mild SA, 1130 as moderate SA, and 974 as severe SA. Subjects with SA were older, were more likely to be male, had higher BMI and prevalence of smoking, drinking, hypertension, diabetes, CHD. There were no statistically significant differences observed among the 4 groups in terms of PIR or the prevalence of race. Regarding the issue of liver injury, as the severity of SA increased, there was a notable and significant rise in the levels of ALT, AST, alkaline phosphatase, gamma glutamyl transpeptidase, and HSI score, as well as the FIB-4 score. Conversely, total protein and albumin exhibited a decrease with SA severity.

**Table 1:** Baseline characteristics and liver injury parameters in subjects stratified by SA severity.

Characteristics	Overall	Absent	Mild	Moderate	Severe	*P*-value
Number of subjects	19,362	15,220	2038	1130	974	
Gender						<.001
Male, number (%)	9320 (47.3%)	7009 (44.9%)	1093 (52.6%)	648 (60.1%)	570 (57.5%)	
Female, number (%)	10,042 (52.7%)	8211 (55.1%)	945 (47.4%)	482 (39.9%)	404 (42.5%)	
Age, years	47.01 ± 18.43	46.10 ± 18.84	48.91 ± 17.13	51.84 ± 16.32	51.56 ± 14.84	<.001
BMI, kg/m^2^	28.75 ± 6.92	28.10 ± 6.63	30.20 ± 7.27	31.05 ± 7.39	32.98 ± 7.44	<.001
Race						.560
Mexican American, number (%)	3034 (7.7%)	2442 (7.8%)	296 (8.1%)	163 (7.8%)	133 (6.5%)	
Other Hispanic, number (%)	1834 (5.6%)	1402 (5.5%)	217 (6.2%)	110 (4.9%)	105 (6.0%)	
Non-Hispanic White, number (%)	7606 (67.2%)	5920 (67.1%)	809 (66.8%)	450 (66.6%)	427 (70.3%)	
Non-Hispanic Black, number (%)	4574 (11.3%)	3614 (11.3%)	481 (11.1%)	268 (11.8%)	211 (9.9%)	
Other race, number (%)	2314 (8.2%)	1842 (8.3%)	235 (7.8%)	139 (9.0%)	98 (7.3%)	
PIR	3.13 ± 1.63	3.13 ± 1.63	3.14 ± 1.62	3.20 ± 1.61	3.09 ± 1.59	.937
Smoking						<.001
Yes, number (%)	6756 (40.3%)	4973 (38.7%)	808 (42.5%)	484 (45.7%)	491 (52.8%)	
No, number (%)	10,448 (59.7%)	8270 (61.3%)	1111 (57.5%)	612 (54.3%)	455 (47.2%)	
Drinking						<.001
Yes, number (%)	13,127 (86.7%)	9895 (85.6%)	1552 (89.3%)	895 (91.2%)	785 (91.0%)	
No, number (%)	2636 (13.3%)	2175 (14.4%)	237 (10.7%)	120 (8.8%)	104 (9.0%)	
Hypertension						<.001
Yes, number (%)	6393 (30.4%)	4562 (27.7%)	796 (36.0%)	520 (40.0%)	515 (48.7%)	
No, number (%)	12,947 (69.6%)	10,641 (72.3%)	1240 (64.0%)	607 (60.0%)	459 (51.3%)	
Diabetes						<.001
Yes, number (%)	2459 (9.9%)	1685 (8.7%)	316 (12.5%)	233 (12.9%)	225 (18.9%)	
No, number (%)	16,903 (90.1%)	13,535 (91.3%)	1722 (87.5%)	897 (87.1%)	749 (81.1%)	
CHD						<.001
Yes, number (%)	802 (4.1%)	540 (3.6%)	103 (4.4%)	78 (6.0%)	81 (7.3%)	
No, number (%)	15,874 (95.9%	12,250 (96.4%)	1768 (95.6%)	1002 (94.0%)	854 (92.7%)	
ALT (U/L)	23.72 ± 16.77	23.08 ± 15.75	25.26 ± 21.65	26.38 ± 17.99	27.21 ± 17.71	<.001
AST (U/L)	23.98 ± 12.55	23.79 ± 12.59	24.43 ± 12.93	24.77 ± 11.70	25.08 ± 11.87	<.001
AST/ALT	1.13 ± 0.35	1.15 ± 0.36	1.08 ± 0.34	1.06 ± 0.35	1.02 ± 0.30	<.001
AKP (U/L)	71.08 ± 25.41	70.88 ± 25.63	71.18 ± 23.48	71.85 ± 25.57	73.02 ± 25.57	.019
TBIL (μmol/L)	10.84 ± 5.79	10.91 ± 5.86	10.41 ± 5.63	10.85 ± 5.40	10.65 ± 5.38	.084
TP (g/dL)	7.11 ± 0.44	7.12 ± 0.44	7.10 ± 0.43	7.08 ± 0.43	7.08 ± 0.43	.001
ALB (g/dL)	4.25 ± 0.34	4.26 ± 0.34	4.25 ± 0.35	4.20 ± 0.33	4.17 ± 0.33	<.001
GGT (U/L)	25.96 ± 34.13	24.62 ± 30.80	27.57 ± 30.93	33.68 ± 63.55	34.30 ± 39.06	<.001
his	1.03 ± 0.79	1.01 ± 0.72	1.09 ± 0.77	1.22 ± 1.58	1.07 ± 0.56	<.001
FIB-4	37.77 ± 8.13	37.01 ± 7.83	39.53 ± 8.38	40.40 ± 8.58	42.72 ± 8.64	<.001

All means and SDs for continuous variables and percentages for categorical variables were weighted, with the exception of the number of participants.

AKP = alkaline phosphatase, ALB = albumin, ALT = alanine aminotransferase, AST = aspartate aminotransferase, BMI = body mass index, CHD = coronary heart disease, FIB-4 = fibrosis-4, GGT = gamma glutamyl transpeptidase, HSI = hepatic steatosis index, PIR = family income-to-poverty ratio, SA = sleep apnea, TBIL = total bilirubin, TP = total protein.

### 3.2. Association between SA and liver injury

Table 2 displays the results of the association between SA and liver injury based on weighted multivariate linear regression. After adjusting for age, gender, race, BMI, PIR, smoking, drinking, hypertension, diabetes, CHD, the presence of SA was significantly associated with lnALT, lnAST/ALT, lnGGT, and lnHSI (all *P* values < .05). In addition, the dose–response relationship between the severity of SA and liver injury was also explored. There is a dose–response relationship between the severity of SA and increased levels of lnALT, lnGGT, decreased level of lnAST/ALT (test for trend, all *P* values < .05). Despite a trend of dose-response relationship was found between SA severity and increased lnHSI, the result did not reach statistically significant differences (test for trend, *P* = .087).

**Table 2:** Associations between SA and liver injury based on weighted multivariate linear regression.

Outcomes		SA status	SA severity			
		SA	Rarely	Moderately	Frequently	*P*-value for trend
LnALT	Adjusted *β* (95% CI)	0.033 (0.008, 0.057)	0.031 (‐0.000, 0.0628)	0.024 (‐0.019, 0.067)	0.044 (0.009, 0.080)	
	*P*-value	0.009	0.052	0.265	0.015	.041
LnAST	Adjusted *β* (95% CI)	0.009 (‐0.007, 0.026)	0.010 (‐0.011, 0.031)	0.006 (‐0.024, 0.035)	0.013 (‐0.022, 0.047)	
	*P*-value	.254	.363	.707	.474	.542
LnAST/ALT	Adjusted β (95% CI)	‐0.023 (‐0.039, ‐0.007)	‐0.021 (‐0.045, 0.002)	‐0.019 (‐0.042, 0.004)	‐0.032 (‐0.055, ‐0.009)	
	*P*-value	.004	.078	.109	.007	.020
lnGGT	Adjusted β (95% CI)	0.058 (0.026, 0.090)	0.021 (‐0.021, 0.063)	0.078 (0.019, 0.136)	0.112 (0.055, 0.170)	
	*P*-value	<.001	.322	.010	<.001	<.001
LnAKP	Adjusted β (95% CI)	-0.008 (-0.020, 0.004)	-0.003 (-0.019, 0.011)	-0.010 (-0.035, 0.014)	-0.013 (-0.038, 0.011)	
	*P*-value	.184	.607	.408	.276	.223
LnTP	Adjusted β (95% CI)	-0.001 (-0.004, 0.001)	-0.001 (-0.005, 0.003)	-0.003 (-0.008, 0.002)	-0.000 (-0.005, 0.004)	
	*P*-value	.317	.577	.197	.869	.654
LnALB	Adjusted β (95% CI)	‐0.000 (‐0.004, 0.004)	0.004 (‐0.001, 0.009)	‐0.005 (‐0.012, 0.002)	‐0.003 (‐0.010, 0.004)	
	*P*-value	.944	.142	.148	.385	.120
LnHSI	Adjusted β (95% CI)	0.006 (0.002, 0.010)	0.006 (0.000, 0.012)	0.005 (-0.001, 0.011)	0.006 (0.000, 0.013)	
	*P*-value	.004	.033	.120	.038	.087
LnFIB-4	Adjusted β (95% CI)	0.011 (‐0.011, 0.032)	0.023 (‐0.005, 0.052)	0.001 (‐0.033, 0.036)	‐0.005 (‐0.044, 0.033)	
	*P*-value	.337	.108	.935	.780	.541

Analyses were adjusted for age, gender, race, BMI, PIR, smoking, drinking, hypertension, diabetes, CHD.

AKP = alkaline phosphatase, ALB = albumin, ALT = alanine aminotransferase, AST = aspartate aminotransferase, CHD = coronary heart disease, FIB-4 = fibrosis-4, GGT = gamma glutamyl transpeptidase, HSI = hepatic steatosis index, SA = sleep apnea, TP = total protein.

### 3.3. Subgroup analyses

**Figure 2.**
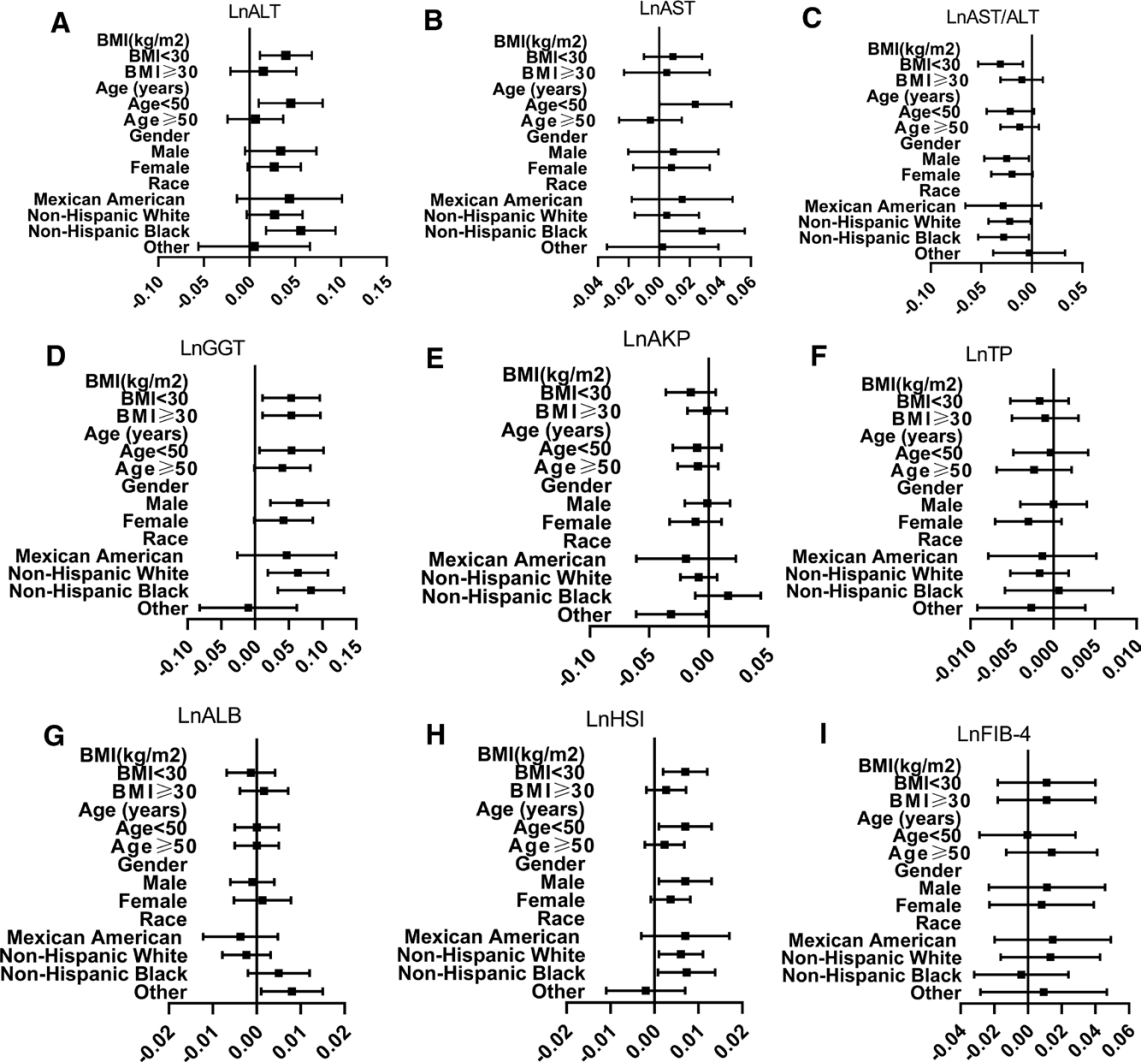
Forest plots of beta coefficients (95% CI) of multivariable linear regression analyses between SA and liver injury in subgroups based on age, BMI, gender, and race. BMI = body mass index, CI = confidence interval, SA = sleep apnea.

The forest plot of adjusted Beta and 95% confidence intervals in subgroups is presented in Figure 2. Subgroup analyses showed that the presence of SA was independently associated with lnALT, lnGGT, lnHSI, and lnAST/ALT in subgroup of BMI < 30 kg/m^2^ (all *P* values < .05). While in BMI ≥ 30, independent association only existed between SA and lnGGT. In subgroup of age < 50 years, SA was found to be independently linked to lnALT, lnAST, lnGGT, and lnHSI (all *P* values < .05). However, no outcome parameter was showed to be independently correlated with SA (all *P* values ≥ .05). SA was identified as having an independent association with lnAST/ALT, lnGGT, and lnHSI in male subjects (all *P* values < .05). In female subjects, there is no observed outcome parameter associated with SA after adjusting confounders (all *P* values > .05). In addition, we found that the association of SA and liver injury were more pronounced among non-Hispanic Black than other races (positive for lnALT, lnAST, lnAST/ALT, lnGGT, and lnHSI). The detailed information of results stratified by subgroups is further summarized in Tables S2, Supplemental Digital Content, http://links.lww.com/MD/N418, S3, Supplemental Digital Content, http://links.lww.com/MD/N420, S4, Supplemental Digital Content, http://links.lww.com/MD/N421, S5, Supplemental Digital Content, http://links.lww.com/MD/N423.

### 3.4. Sensitivity analysis

Table 3 shows results of a sensitivity analysis that was conducted after imputing missing data for confounding variables. SA was significantly correlated with lnALT, lnAST/ALT, lnGGT, and lnHSI after adjusting confounders. A dose–response relationship between the severity of SA and lnALT, lnGGT, lnAST/ALT was noted. The results of analysis for imputed data were similar to the data without imputing missing values, which confirmed the robust results.

**Table 3:** Results of weighted multivariate linear regression after imputing data for confounding variables.

Outcomes		SA status	SA severity			
		SA	Rarely	Moderately	Frequently	*P*-value for trend
LnALT	Adjusted *β* (95% CI)	0.033 (0.013, 0.054)	0.031 (‐0.000, 0.0628)	0.028 (‐0.012, 0.069)	0.054 (0.022, 0.085)	
	*P*-value	.001	.068	.168	.001	.006
LnAST	Adjusted *β* (95% CI)	0.007 (-0.007, 0.021)	0.004 (-0.015, 0.023)	0.003 (-0.024, 0.030)	0.017 (-0.014, 0.049)	
	*P*-value	.336	.674	.825	.279	.336
LnAST/ALT	Adjusted β (95% CI)	-0.026 (-0.041, -0.012)	-0.023 (-0.044, -0.001)	-0.025 (-0.047, -0.003)	-0.036 (-0.057, -0.016)	
	*P*-value	<.001	.037	.024	<.001	.003
lnGGT	Adjusted β (95% CI)	0.058 (0.031, 0.085)	0.022 (-0.012, 0.057)	0.078 (0.025, 0.131)	0.112 (0.061, 0.162)	
	*P*-value	<.001	.204	.005	<.001	<.001
LnAKP	Adjusted β (95% CI)	‐0.011 (‐0.023, 0.001)	‐0.008 (‐0.025, 0.009)	‐0.014 (‐0.036, 0.007)	‐0.012 (‐0.034, 0.010)	
	*P*-value	.080	.334	.188	.283	.244
LnTP	Adjusted β (95% CI)	‐0.001 (‐0.004, 0.002)	0.000 (‐0.004, 0.003)	‐0.002 (‐0.006, 0.003)	‐0.001 (‐0.006, 0.003)	
	*P*-value	.499	.577	.443	.584	.477
LnALB	Adjusted β (95% CI)	0.000 (‐0.004, 0.003)	0.003 (‐0.001, 0.008)	‐0.005 (‐0.011, 0.001)	‐0.003 (‐0.009, 0.003)	
	*P*-value	.827	.135	.118	.260	.069
LnHSI	Adjusted β (95% CI)	0.006 (0.003, 0.010)	0.006 (0.001, 0.012)	0.006 (0.000, 0.012)	0.007 (0.001, 0.012)	
	*P*-value	<.001	.014	.036	.026	.050
LnFIB-4	Adjusted β (95% CI)	0.008 (‐0.011, 0.027)	0.016 (‐0.008, 0.040)	‐0.002 (‐0.033, 0.028)	0.001 (‐0.030, 0.033)	
	*P*-value	.412	.190	.878	.934	.775

Analyses were adjusted for age, gender, race, BMI, PIR, smoking, drinking, hypertension, diabetes, CHD.

AKP = alkaline phosphatase, ALB = albumin, ALT = alanine aminotransferase, AST = aspartate aminotransferase, CHD = coronary heart disease, FIB-4 = fibrosis-4, GGT = gamma glutamyl transpeptidase, HSI = hepatic steatosis index, SA = sleep apnea, TP = total protein.

## 4. Discussion

The present study suggested that self-reported SA was independently associated with elevated liver enzymes and aggravated liver steatosis, but not with liver fibrosis. A dose–response relationship between severity of SA and elevated liver enzymes was also noted. Subgroup analyses revealed that the association of SA and liver injury was more pronounced among nonobese, younger, non-Hispanic Black, and male populations.

The relationship between OSA and liver injury has been investigated for many years. A study demonstrated an independent correlation between the severity of nocturnal hypoxia and the presence of steatosis assessed by noninvasive blood tests.^[14]^ Trzepizur et al^[15]^ studied 147 patients with metabolic comorbidities and found that severe OSA was associated with increased liver stiffness after controlling confounding factors. Another small-sample study focusing pediatric population showed significant correlations between OSA-related hypoxia and elevated transaminases, hepatic steatosis, NAFLD inflammation score, and liver fibrosis stage.^[16]^ Despite the strong evidence supporting the impact of OSA on liver injury, some other studies have not found any association between them.^[17,18]^ Daltro et al^[17]^ conducted a study on 40 obese patients and revealed that OSA-related parameters did not show significant correlations with transaminases, liver histopathological characteristics, or nonalcoholic steatohepatitis.

Most studies only focused on obese patients in hospital and were limited by small sample size. Furthermore, the results remained controversial. Hence, this study with large sample size was performed based on community population. Our results supported an independent relationship between SA and elevated liver enzymes, aggravated liver steatosis. But this was not case for liver fibrosis. Consistent with the results of the present study, a multisite cross-sectional study with 1285 subjects evaluating OSA severity and blood markers of liver injury reported that OSA severity and sleep-related hypoxemia were independently associated with liver steatosis and liver cytolysis, but not with significant liver fibrosis.^[13]^ In one of our previous studies, we included 160 consecutive patients suspected for OSA and identified that OSA severity was independently associated with liver steatosis and elevation of serum aminotransferases, but not with liver fibrosis.^[19]^ However, a meta-analysis showed that OSA was associated with increased risk of liver fibrosis independently of age, gender, and BMI.^[20]^ The discrepancy of results can be explained by the difference in the study samples. The majority of the studies included in the meta-analysis focused on morbidly obese patients undergoing bariatric surgery with intraoperative liver biopsies or patients with abnormal liver enzymes.

Several mechanisms contribute to the association between SA and elevated liver enzymes and aggravated liver steatosis. CIH triggers systemic inflammation, oxidative stress, and metabolic dysregulation, including insulin resistance and dyslipidemia.^[21]^ These factors lead to hepatic inflammation, lipid accumulation, and oxidative damage. Additionally, CIH induces endoplasmic reticulum stress and even liver cell ferroptosis.^[22,23]^

Some clinical implications for the results could be highlighted. Based on the findings of the present study, it is suggested that it was necessary to closely monitor the liver injury for SA population, especially in nonobese, younger, non-Hispanic Black, and male subjects. In addition, some evidences have demonstrated a beneficial effect of OSA treatment on liver injury in adults^[24]^ and pediatric population.^[25]^ This emphasizes the significance of providing OSA treatments for these patients.

Subgroup analyses revealed that the links between SA and liver injury were more pronounced in nonobese, younger, non-Hispanic Black, and male population. In line with the results of the present study, we previously found that severe OSA was positively correlated with liver injury in males, while female OSA was not associated with liver injury in a group of Chinese Han population from the hospital.^[26]^ Furthermore, another American study also found that the relationship between OSA and cardiovascular risk factors were stronger in male, younger individuals, and African American individuals.^[27]^ Similar to above results, Ghazi and coworkers^[28]^ reported high risk of OSA was correlated with prevalent atrial fibrillation among black people but not white people. These findings reflected differences in population susceptibility to OSA-related multiple organ injury. Further investigation of inter-individual differences in responses to OSA-related stressors or the genetic susceptibility may help identify targets for future interventions.

Our study possesses several notable strengths. Firstly, to the best of our knowledge, this study represents the largest sample size to date investigating the relationship between SA and liver injury. The large sample size allows us to establish a more robust conclusion. Secondly, this study used a community-based sample, thereby providing a better reflection of the general population compared to clinically-based studies. Thirdly, subgroup analyses were performed in this study, which provides insight into the variation in the relationship between OSA and liver injury across groups, assisting in the identification of the appropriate treatment population. Finally, the sensitivity analysis revealed that the conclusions remained robust even when subjected to methodological changes, indicating the reliability of the data in the present study.

Despite the strengths, some limitations should be considered. Firstly, the cross-sectional study design dose not allows us to establish causality between SA and liver injury. Secondly, the assessment of SA was solely based on self-reporting rather than objective indicators such as polysomnography. There may be both overestimation and underestimation of the true prevalence and severity of SA in our study population. However, self-reported data provide a feasible and cost-effective means for large-scale epidemiological studies. Our findings align with existing literature, further supporting the validity of our results. Thirdly, the diagnostic indices used in our study, namely the HSI and FIB-4, are not considered as the gold standards for diagnosing liver steatosis and liver fibrosis. However, previous studies have demonstrated a good performance of HSI and FIB-4 in diagnosing liver steatosis and liver fibrosis,^[29,30]^ respectively. Finally, despite excluding participants with excessive alcohol intake, over 85% of the study population still drinks alcohol, which could potentially influence the results.

In conclusion, our findings indicated significant association between self-reported SA and elevated liver enzymes, aggravated liver steatosis among US population. The association was more pronounced among nonobese, younger, non-Hispanic black, and male subgroups. The results of our study suggested that it might be prudent to monitor the liver injury for people with SA.

## Author contributions

**Conceptualization:** Zhi-Wei Huang.

**Data curation:** Yi-Bin Jiang.

**Formal analysis:** Xue-Jun Lin, Li-Da Chen.

**Investigation:** Yi-Bin Jiang, Zhi-Wei Huang.

**Methodology:** Yi-Bin Jiang, Li-Da Chen.

**Project administration:** Zhi-Wei Huang.

**Software:** Jia-Min Luo.

**Writing – original draft:** Yi-Bin Jiang.

**Writing – review & editing:** Zhi-Wei Huang, Li-Da Chen.

## Supplementary Material


