# Supplementary material for: Association between self-reported sleep apnea and biomarkers of liver injury: Evidence from National Health and Nutrition Examination Survey
Source: Medicine (Baltimore). 2024 Sep 6;103(36):e39393. doi: 10.1097/MD.0000000000039393 (PMC12431730; doi:10.1097/MD.0000000000039393)
Supplement: Supplementary file 1 [file medi-103-e39393-s001.docx]

Table S1 The numbers and percentages of missing covariate data

| Covariate | Numbers | Percentages (%) |
| --- | --- | --- |
| BMI | 302 | 1.6 |
| CHD | 2686 | 13.9 |
| Hypertension | 22 | 0.1 |
| Smoking | 2158 | 11.1 |
| Drinking | 3599 | 18.6 |
| PIR | 1868 | 9.6 |

Abbreviation: BMI=body mass index, CHD=coronary heart disease, PIR=family income-to-poverty ratio.
