# Supplementary material for: Association between self-reported sleep apnea and biomarkers of liver injury: Evidence from National Health and Nutrition Examination Survey
Source: Medicine (Baltimore). 2024 Sep 6;103(36):e39393. doi: 10.1097/MD.0000000000039393 (PMC12431730; doi:10.1097/MD.0000000000039393)
Supplement: Supplementary file 2 [file medi-103-e39393-s002.docx]

Table S2 Subgroup analyses based on BMI

| Outcomes | SA status | |
| --- | --- | --- |
|  | Adjusted β (95%CI) | p value |
| **LnALT** |  |  |
| BMI < 30 (N=8,285) | 0.040 (0.011, 0.068) | 0.005 |
| BMI ≥ 30 (N=5,408) | 0.015 (-0.021, 0.051) | 0.405 |
| **LnAST** |  |  |
| BMI < 30 (N=8,285) | 0.009 (-0.010, 0.028) | 0.347 |
| BMI ≥ 30 (N=5,408) | 0.005 (-0.023, 0.033) | 0.707 |
| **LnAST/ALT** |  |  |
| BMI < 30 (N=8,285) | -0.031 (-0.053, -0.009) | 0.005 |
| BMI ≥ 30 (N=5,408) | -0.010 (-0.031, 0.011) | 0.354 |
| **LnGGT** |  |  |
| BMI < 30 (N=8,285) | 0.054 (0.011, 0.096) | 0.011 |
| BMI ≥ 30 (N=5,408) | 0.054 (0.011, 0.097) | 0.012 |
| **LnAKP** |  |  |
| BMI < 30 (N=8,285) | -0.015 (-0.036, 0.006) | 0.154 |
| BMI ≥ 30 (N=5,408) | -0.001 (-0.018, 0.015) | 0.866 |
| **LnTP** |  |  |
| BMI < 30 (N=8,285) | -0.002 (-0.005, 0.002) | 0.396 |
| BMI ≥ 30 (N=5,408) | -0.001 (-0.005, 0.003) | 0.637 |
| **LnALB** |  |  |
| BMI < 30 (N=8,285) | -0.001 (-0.007, 0.004) | 0.663 |
| BMI ≥ 30 (N=5,408) | 0.002 (-0.004, 0.007) | 0.564 |
| **LnHSI** |  |  |
| BMI < 30 (N=8,285) | 0.007 (0.002, 0.012) | 0.006 |
| BMI ≥ 30 (N=5,408) | 0.003 (-0.002, 0.007) | 0.263 |
| **LnFIB-4** |  |  |
| BMI < 30 (N=8,285) | 0.011 (-0.018, 0.040) | 0.444 |
| BMI ≥ 30 (N=5,408) | 0.011 (-0.018, 0.040) | 0.445 |

Analyses were adjusted for age, gender, race, BMI, PIR, smoking, drinking, hypertension, diabetes, CHD.

Abbreviation: ALT=alanine aminotransferase, AST=aspartate aminotransferase, AKP= alkaline phosphatase, TP=total protein, ALB=albumin, GGT=gamma glutamyl transpeptidase, HSI= hepatic steatosis index, FIB-4= fibrosis-4.
