# Supplementary material for: Association between self-reported sleep apnea and biomarkers of liver injury: Evidence from National Health and Nutrition Examination Survey
Source: Medicine (Baltimore). 2024 Sep 6;103(36):e39393. doi: 10.1097/MD.0000000000039393 (PMC12431730; doi:10.1097/MD.0000000000039393)
Supplement: Supplementary file 3 [file medi-103-e39393-s003.docx]

Table S3 Subgroup analyses based on age

| Outcomes | SA status | |
| --- | --- | --- |
|  | Adjusted β (95%CI) | p value |
| **LnALT** |  |  |
| Age < 50 (N=6,123) | 0.045 (0.010, 0.080) | 0.010 |
| Age ≥ 50 (N=7,570) | 0.006 (-0.024, 0.037) | 0.674 |
| **LnAST** |  |  |
| Age < 50 (N=6,123) | 0.024 (0.000, 0.047) | 0.042 |
| Age ≥ 50 (N=7,570) | -0.006 (-0.026, 0.015) | 0.582 |
| **LnAST/ALT** |  |  |
| Age < 50 (N=6,123) | -0.021 (-0.045, 0.002) | 0.071 |
| Age ≥ 50 (N=7,570) | -0.012 (-0.031, 0.007) | 0.205 |
| **LnGGT** |  |  |
| Age < 50 (N=6,123) | 0.054 (0.007, 0.102) | 0.022 |
| Age ≥ 50 (N=7,570) | 0.041 (-0.001, 0.082) | 0.050 |
| **LnAKP** |  |  |
| AGE < 50 (N=6,123) | -0.010 (-0.030, 0.011) | 0.349 |
| AGE ≥ 50 (N=7,570) | -0.009 (-0.026, 0.008) | 0.293 |
| **LnTP** |  |  |
| Age < 50 (N=6,123) | 0.000 (-0.005, 0.004) | 0.816 |
| Age ≥ 50 (N=7,570) | -0.002 (-0.007, 0.002) | 0.355 |
| **LnALB** |  |  |
| Age < 50 (N=6,123) | 0.000 (-0.005, 0.005) | 0.909 |
| Age ≥ 50 (N=7,570) | 0.000 (-0.005, 0.005) | 0.983 |
| **LnHSI** |  |  |
| Age < 50 (N=6,123) | 0.007 (0.001, 0.013) | 0.030 |
| Age ≥ 50 (N=7,570) | 0.002 (-0.002, 0.007) | 0.349 |
| **LnFIB-4** |  |  |
| Age < 50 (N=6,123) | 0.000 (-0.029, 0.028) | 0.976 |
| Age ≥ 50 (N=7,570) | 0.014 (-0.013, 0.041) | 0.293 |

Analyses were adjusted for age, gender, race, BMI, PIR, smoking, drinking, hypertension, diabetes, CHD.

Abbreviation: ALT=alanine aminotransferase, AST=aspartate aminotransferase, AKP= alkaline phosphatase, TP=total protein, ALB=albumin, GGT=gamma glutamyl transpeptidase, HSI= hepatic steatosis index, FIB-4= fibrosis-4.
