# Supplementary material for: Association between self-reported sleep apnea and biomarkers of liver injury: Evidence from National Health and Nutrition Examination Survey
Source: Medicine (Baltimore). 2024 Sep 6;103(36):e39393. doi: 10.1097/MD.0000000000039393 (PMC12431730; doi:10.1097/MD.0000000000039393)
Supplement: Supplementary file 4 [file medi-103-e39393-s004.docx]

Table S4 Subgroup analyses based on gender

| Outcomes | SA status | |
| --- | --- | --- |
|  | Adjusted β (95%CI) | p value |
| **LnALT** |  |  |
| Male (N= 6,609) | 0.034 (-0.005, 0.073) | 0.081 |
| Female (N=7,084) | 0.027 (-0.002, 0.056) | 0.058 |
| **LnAST** |  |  |
| Male (N= 6,609) | 0.009 (-0.020, 0.039) | 0.519 |
| Female (N=7,084) | 0.008 (-0.017, 0.033) | 0.544 |
| **LnAST/ALT** |  |  |
| Male (N= 6,609) | -0.025 (-0.047, -0.003) | 0.026 |
| Female (N=7,084) | -0.020 (-0.040, 0.001) | 0.060 |
| **LnGGT** |  |  |
| Male (N=6,609) | 0.066 (0.023, 0.109) | 0.002 |
| Female (N=7,084) | 0.042 (-0.001, 0.086) | 0.052 |
| **LnAKP** |  |  |
| Male (N=6,609) | -0.001 (-0.020, 0.018) | 0.907 |
| Female (N=7,084) | -0.011 (-0.033, 0.011) | 0.326 |
| **LnTP** |  |  |
| Male (N=6,609) | 0.000 (-0.004, 0.004) | 0.966 |
| Female (N=7,084) | -0.003 (-0.007, 0.001) | 0.152 |
| **LnALB** |  |  |
| Male (N=6,609) | -0.001 (-0.006, 0.004) | 0.776 |
| Female (N=7,084) | 0.001 (-0.005, 0.008) | 0.749 |
| **LnHSI** |  |  |
| Male (N=6,609) | 0.007 (0.001, 0.013) | 0.020 |
| Female (N=7,084) | 0.004 (-0.001, 0.008) | 0.093 |
| **LnFIB-4** |  |  |
| Male (N=6,609) | 0.011 (-0.023, 0.046) | 0.512 |
| Female (N=7,084) | 0.008 (-0.023, 0.039) | 0.602 |

Analyses were adjusted for age, gender, race, BMI, PIR, smoking, drinking, hypertension, diabetes, CHD.

Abbreviation: ALT=alanine aminotransferase, AST=aspartate aminotransferase, AKP= alkaline phosphatase, TP=total protein, ALB=albumin, GGT=gamma glutamyl transpeptidase, HSI= hepatic steatosis index, FIB-4= fibrosis-4.
