# Supplementary material for: Association between self-reported sleep apnea and biomarkers of liver injury: Evidence from National Health and Nutrition Examination Survey
Source: Medicine (Baltimore). 2024 Sep 6;103(36):e39393. doi: 10.1097/MD.0000000000039393 (PMC12431730; doi:10.1097/MD.0000000000039393)
Supplement: Supplementary file 5 [file medi-103-e39393-s005.docx]

Table S5 Subgroup analyses based on race

| Outcomes | SA status | |
| --- | --- | --- |
|  | Adjusted β (95%CI) | p value |
| **LnALT** |  |  |
| Mexican American (N= 1,867) | 0.044 (-0.014, 0.101) | 0.130 |
| Non-Hispanic White (N=5,958) | 0.027 (-0.003, 0.058) | 0.074 |
| Non-Hispanic Black (N=3,105) | 0.056 (0.018, 0.094) | 0.003 |
| Other (N=2,763) | 0.005 (-0.056, 0.066) | 0.864 |
| **LnAST** |  |  |
| Mexican American (N= 1,867) | 0.015 (-0.018, 0.048) | 0.352 |
| Non-Hispanic White (N=5,958) | 0.005 (-0.016, 0.026) | 0.631 |
| Non-Hispanic Black (N=3,105) | 0.028 (0.000, 0.056) | 0.047 |
| Other (N=2,763) | 0.002 (-0.034, 0.039) | 0.897 |
| **LnAST/ALT** |  |  |
| Mexican American (N= 1,867) | -0.028 (-0.066, 0.009) | 0.130 |
| Non-Hispanic White (N=5,958) | -0.022 (-0.043, -0.001) | 0.033 |
| Non-Hispanic Black (N=3,105) | -0.028 (-0.053, -0.003) | 0.023 |
| Other (N=2,763) | -0.003 (-0.038, 0.033) | 0.871 |
| **LnGGT** |  |  |
| Mexican American (N= 1,867) | 0.047 (-0.026, 0.120) | 0.195 |
| Non-Hispanic White (N=5,958) | 0.064 (0.019, 0.108) | 0.004 |
| Non-Hispanic Black (N=3,105 ) | 0.083 (0.034, 0.132) | <0.001 |
| Other (N=2,763) | -0.010 (-0.082, 0.062) | 0.783 |
| **LnAKP** |  |  |
| Mexican American (N= 1,867) | -0.019 (-0.061, 0.023) | 0.364 |
| Non-Hispanic White (N=5,958) | -0.008 (-0.024, 0.007) | 0.279 |
| Non-Hispanic Black (N=3,105 ) | 0.016 (-0.011, 0.044) | 0.232 |
| Other (N=2,763) | -0.032 (-0.061, -0.002) | 0.030 |
| **LnTP** |  |  |
| Mexican American (N= 1,867) | -0.001 (-0.008, 0.005) | 0.632 |
| Non-Hispanic White (N=5,958) | -0.002 (-0.005, 0.002) | 0.329 |
| Non-Hispanic Black (N=3,105 ) | 0.001 (-0.006, 0.007) | 0.840 |
| Other (N=2,763) | -0.003 (-0.009, 0.004) | 0.392 |
| **LnALB** |  |  |
| Mexican American (N= 1,867) | -0.004 (-0.012, 0.005) | 0.374 |
| Non-Hispanic White (N=5,958) | -0.002 (-0.008, 0.003) | 0.429 |
| Non-Hispanic Black (N=3,105 ) | 0.005 (-0.002, 0.012) | 0.167 |
| Other (N=2,763) | 0.008 (0.001, 0.015) | 0.031 |
| **LnHSI** |  |  |
| Mexican American (N= 1,867) | 0.007 (-0.003, 0.017) | 0.142 |
| Non-Hispanic White (N=5,958) | 0.006 (0.001, 0.011) | 0.022 |
| Non-Hispanic Black (N=3,105 ) | 0.007 (0.001, 0.014) | 0.018 |
| Other (N=2,763) | -0.002 (-0.011, 0.007) | 0.658 |
| **LnFIB-4** |  |  |
| Mexican American (N= 1,867) | 0.015 (-0.020, 0.049) | 0.384 |
| Non-Hispanic White (N=5,958) | 0.013 (-0.016, 0.043) | 0.365 |
| Non-Hispanic Black (N=3,105 ) | -0.004 (-0.032, 0.024) | 0.783 |
| Other (N=2,763) | 0.009 (-0.028, 0.047) | 0.613 |

Analyses were adjusted for age, gender, race, BMI, PIR, smoking, drinking, hypertension, diabetes, CHD.

Abbreviation: ALT=alanine aminotransferase, AST=aspartate aminotransferase, AKP= alkaline phosphatase, TP=total protein, ALB=albumin, GGT=gamma glutamyl transpeptidase, HSI= hepatic steatosis index, FIB-4= fibrosis-4.
